# Preparation and Infection of Bovine Precision‐Cut Lung Slices (b‐PCLS) as an *Ex Vivo* Model for Respiratory Pathogens

**DOI:** 10.1002/cpz1.70459

**Published:** 2026-09-24

**Authors:** Asal Ahmadi, Ghazal Ahmadi, Vilde Galleberg‐Wahlberg, Abdolrahman Khezri, Veslemøy Sunniva Oma, Jochen Meens, Mette Myrmel, Ann‐Katrin Llarena

**Affiliations:** ^1^ Department of Paraclinical Sciences, Faculty of Veterinary Medicine Norwegian University of Life Sciences, Ås Norway; ^2^ Department of Biotechnology, Faculty of Ecology, Agriculture, and Biotechnology University of Inland Norway Hamar Norway; ^3^ Department of Production Animal Clinical Sciences, Faculty of Veterinary Medicine Norwegian University of Life Sciences, Ås Norway; ^4^ Institute of Microbiology University of Veterinary Medicine Hannover Hannover Germany

**Keywords:** bovine respiratory disease (BRD), bovine precision‐cut lung slices (b‐PCLS), *ex vivo* infection model, RNA extraction, *Pasteurella multocida*, bovine coronavirus

## Abstract

Precision‐cut lung slices (PCLS) represent a physiologically relevant *ex vivo* model that bridges the gap between simplified *in vitro* systems and *in vivo* animal studies by preserving native lung architecture, cellular diversity, and functional responses. This model provides a valuable platform for investigating respiratory tract infections and tissue‐level host–pathogen interactions in bovine lung tissue, including those involving pathogens associated with bovine respiratory disease (BRD), a leading cause of morbidity, mortality, and economic loss in the global cattle industry. While PCLS protocols are well established for murine and human lungs, standardized and detailed methodologies optimized for bovine lung tissue remain scarce. Here, we present a comprehensive, reproducible protocol for generating, culturing, assessing viability, inoculating, and extracting RNA from bovine precision‐cut lung slices (b‐PCLS). The workflow includes lung inflation with 1.5% low‐melting‐point agarose, vibratome slicing, and culture conditions that preserve bronchiolar architecture and ciliary activity. Viability is assessed longitudinally using ciliary activity scoring and a metabolic assay, demonstrating that b‐PCLS remain viable and highly active for at least 7 days in culture. We also describe inoculation procedures applicable to bacterial and viral pathogens relevant to BRD, exemplified by *Pasteurella multocida* and bovine coronavirus (BCoV), along with standardized sampling strategies for downstream analyses. Finally, we provide an optimized RNA extraction protocol that yields high‐quality RNA from both control and inoculated b‐PCLS, supporting downstream host and pathogen transcriptomic analyses. Collectively, these protocols expand the applicability of large‐animal PCLS for research on respiratory infections, support the principles of replacement and reduction in animal experimentation, and provide a complementary platform for controlled investigation of tissue‐level host–pathogen interactions in bovine lungs. Overall, this *ex vivo* system offers a valuable tool for bovine respiratory research, assay development, and molecular analysis. © 2026 The Author(s). *Current Protocols* published by Wiley Periodicals LLC.

**Basic Protocol 1**: Generation, maintenance, and viability assessment of b‐PCLS

**Basic Protocol 2**: Inoculation of b‐PCLS with *Pasteurella multocida*

**Basic Protocol 3**: Inoculation of b‐PCLS with bovine coronavirus

**Basic Protocol 4**: Extraction of RNA from culture supernatants and b‐PCLS

## Introduction

Lower respiratory tract infections, including pneumonia, are among the top ten causes of death in humans worldwide (World Health Organization, 2021). Beyond human health, respiratory diseases are also a major cause of morbidity, mortality, and welfare concerns in animals, resulting in substantial economic losses (Storoni et al., 2026). In cattle, bovine respiratory disease (BRD) remains a global challenge despite the availability of vaccines and antimicrobial treatments (McAllister et al., 2024). Understanding the molecular mechanisms underlying the pathogenesis of multifactorial and polymicrobial respiratory diseases, such as BRD, is therefore essential for developing targeted, novel control strategies to ensure animal health and welfare and reduce antimicrobial resistance.

Investigations of respiratory infections in humans and animals have traditionally relied on *in vitro* cell culture systems and *in vivo* animal models. These approaches have provided valuable insights into pathogen replication, immune signaling, and host responses. However, important limitations remain. Conventional two‐dimensional (2D) *in vitro* models fail to recapitulate the complex three‐dimensional (3D) architecture, cellular heterogeneity, and multicellular interactions of the lung. This limits their physiological relevance and translational value compared to more complex systems (Yin et al., 2025). Advanced 3D *in vitro* models, including airway epithelial cells cultured at the air‐liquid interface (ALI culture), which form mucociliary pseudostratified epithelium and facilitate airborne pathogen exposures (Cao et al., 2021; Weldearegay et al., 2025); lung organoids, which self‐organize into 3D structures closely resembling native lung tissue (Nikolić & Rawlins, 2017); and lung‐on‐chip systems, which incorporate multicellular tissue environments with electronic monitoring under flow conditions, are increasingly applied in respiratory research (Gkatzis et al., 2018). Each of these methods presents specific challenges, such as long production times and careful handling requirements (ALI culture), the need for sophisticated culture conditions and partial disruption of the natural architecture for infection (organoids), and complex setup and validation procedures (lung‐on‐chip) (Eymieux et al., 2024; Gkatzis et al., 2018). On the other hand, *in vivo* models, while providing physiological complexity, are resource‐intensive, costly, and constrained by ethical considerations (Zscheppang et al., 2018). Moreover, surrogate animal models, such as mice, may not accurately reflect host‐pathogen interactions relevant to the natural host. For instance, evaluating the virulence of bovine respiratory bacterial isolates such as *P. multocida* in mice may not capture the same pathogenic mechanisms or host responses that occur in cattle, the natural host species. Studying these interactions directly in bovine lung tissue is therefore more likely to yield biologically relevant insights into BRD pathomechanisms. Together, these limitations highlight the need for more specialized models that closely mirror the biology of the target host and its natural pathogens.

*Ex vivo* models have emerged as an important intermediate between *in vitro* and *in vivo* approaches by providing a more realistic tissue environment while reducing animal use in accordance with the principles of replacement, reduction, and refinement (3Rs) (Carranza‐Rosales et al., 2017; Crue et al., 2023; Liu et al., 2015; Temann et al., 2017; European Union, 2010). Several *ex vivo* respiratory tissue models have been described, including nasal mucosa explants (NME) and tracheal organ cultures (TOC), which preserve aspects of the respiratory epithelium but are generally limited to specific regions of the upper airway. Precision‐cut lung slices (PCLS), in contrast, preserve native lung architecture, including both bronchiolar and alveolar compartments, extracellular matrix composition, cellular diversity, tissue‐level interactions, and functional responses, including ciliary activity and innate immune signaling (Alsafadi et al., 2020; Bargmann et al., 2025; Eymieux et al., 2024; Gaudino et al., 2023; Koziol‐White et al., 2024; Michalaki et al., 2022; Tatcheff et al., 2025). PCLS are uniform‐thickness lung tissue slices that can be generated in large numbers from a single animal, enabling multiple experimental conditions and technical replicates to be tested simultaneously (Viana et al., 2022). Compared with *in vivo* models, PCLS can reduce overall animal use and, once established, provide a practical, cost‐effective, and ethically favorable *ex vivo* platform for studying lung pathophysiology and pharmacological responses. Viable PCLS can be maintained for several days, allowing investigations of bacterial adherence, invasion, viral replication, and host‐pathogen transcriptional responses without the ethical burden associated with live‐animal experimentation (Bryson et al., 2020; Michalaki et al., 2022).

PCLS have been widely applied in infectious disease research, including studies of bacterial, fungal, and viral infections (Carranza‐Rosales et al., 2017; Fu et al., 2018; Gaudino et al., 2023; Gonzales‐Huerta et al., 2024; Graham et al., 2016; Kirchhoff et al., 2014; Krimmling & Schwegmann‐Weßels, 2017; Molina‐Torres et al., 2020; Munyonho et al., 2024; Qin et al., 2021; Remot et al., 2021; Vötsch et al., 2021; Weldearegay et al., 2025). Experimental endpoints can range from transcriptomic, proteomic, and metabolomic analyses to imaging‐based assessments of cellular dynamics (Akram et al., 2019; Eymieux et al., 2024; Khan et al., 2021). While PCLS protocols are well established for murine and human lung tissue, and porcine PCLS have been described in several studies, standardized and optimized methodologies for bovine PCLS remain limited (Brügger et al., 2025; Crue et al., 2023; Eymieux et al., 2024; Gaudino et al., 2023; H. Zhang et al., 2025; Michalaki et al., 2022; Qin et al., 2021; Remot et al., 2021; Tatcheff et al., 2025; Vötsch et al., 2021; Weldearegay et al., 2025; Weldearegay et al., 2019). This represents a critical gap, as BRD is a major burden in cattle, and host‐specific tissue responses are important for better understanding the molecular mechanisms involved in pathogenesis and therapeutic efficacy. Importantly, b‐PCLS can be generated from cattle slaughtered for food production, enabling the use of animal by‐products and supporting the principles of reduction and replacement in animal research. While b‐PCLS are best regarded as a controlled platform for dissecting discrete components of host–pathogen interaction at the tissue level, rather than as a complete model of disease in the field, they retain resident immune cell populations, including alveolar macrophages, dendritic cells, and T cells that are capable of mounting immune responses to pathogen challenge (Crue et al., 2023; Koziol‐White et al., 2024; Liu et al., 2019; Viana et al., 2022). Furthermore, the transcriptomic and cytokine profiling of b‐PCLS mono‐ and co‐infections are directly compatible with and build upon existing knowledge of immune and cytokine interactions in respiratory diseases, such as BRD — in which viral pathogens like bovine respiratory syncytial virus (BRSV) or BCoV typically precede bacterial infection (Ackermann et al., 2010; Gaudino et al., 2023; Remot et al., 2021; Weldearegay et al., 2019). This provides mechanistic insight at the tissue level that complements, rather than replaces, *in vivo* and field‐based immunological studies. In addition, b‐PCLS may serve as a valuable large‐animal model for comparative respiratory research beyond BRD, with potential translational applications in pulmonary toxicology, pharmacology, and One Health research (Eymieux et al., 2024; Koziol‐White et al., 2024; Liu et al., 2019; Prohl et al., 2014). A range of PCLS applications is listed in Table 1. The interpretive limitations of the model are also discussed in the Critical Parameters section.

**Table 1:** Key Applications of precision‐cut lung slices (PCLS) in respiratory research

Application area	Representative readouts	Relevance/advantage
**Physiology & pathophysiology**	Ciliary dynamics, airway contractility, vasoconstriction and dilation (live imaging) Histological and ultrastructural assessment (imaging) Metabolic viability (resazurin assays) Applications in fibrosis, chronic obstructive pulmonary disease (COPD), asthma, allergic responses, lung cancer research (Blomberg et al., 2024; Koziol‐White et al., 2024; Lauenstein et al., 2014; Lehmann et al., 2025)	Preservation of native structural features: bronchiolar and alveolar compartments, extracellular matrix (collagen, fibronectin, elastin), basement membrane, and tissue geometry Cellular diversity: ciliated and non‐ciliated epithelial cells, goblet cells and club (Clara) cells, type I and type II pneumocytes, alveolar macrophages and dendritic cells, fibroblasts, endothelial cells, smooth muscle cells, mast cells, NK and T cell subsets. Functional features: ciliary beating, innate immune signaling, tissue injury and repair Possibility to cryopreserve for future use (Bargmann et al., 2025; Crue et al., 2023; Eymieux et al., 2024; Koziol‐White et al., 2024)
**Infection biology**	Bacterial adherence, invasion, biofilm research Viral amplification (RT‐qPCR) Host & pathogen gene expression (RT‐qPCR, RNA‐seq) Cytokine & chemokine release profiling (ELISA, multiplex) Pathogen tropism (confocal and fluorescence microscopy) Histopathological changes over time (H&E) Cytotoxicity & tissue integrity (resazurin assays, LDH release assays) (Furnica et al., 2025; Gaudino et al., 2023; Gonzales‐Huerta et al., 2024; Munyonho et al., 2024; Remot et al., 2021; Vötsch et al., 2021; Weldearegay et al., 2025)	Controlled exposure of lung tissue to bacterial, viral, and fungal respiratory pathogens (BSL‐2 and BSL‐3) under defined experimental conditions in native host lung tissue Supports mechanistic investigation of tissue‐level host‐pathogen interactions Captures pathogen replication dynamics & epithelial tropism Compatible with sequential co‐infection designs
**Immunology**	Identification and quantification of resident immune cell populations after enzymatic tissue dissociation (flow cytometry, CyTOF) Cell surface marker profiling Apoptosis and intracellular signaling markers Spatial localization of immune cells by confocal microscopy (Bargmann et al., 2025; Koziol‐White et al., 2024)	Enables characterization of resident lung immune cell populations
**Transcriptomics & other relevant omics**	RNA yield and integrity RT‐qPCR for host and pathogen targets Bulk RNA‐seq & differential expression analysis Compatible with downstream proteomic & metabolomic analyses (Khan et al., 2021)	Recovery of high‐quality RNA suitable for simultaneous profiling of host and pathogen transcriptomes from the same experimental material; supports comprehensive molecular characterization of infection responses
**Pharmacology**	Pathogen burden (RT‐qPCR, CFU counts) Tissue viability under treatment (resazurin assays, LDH release assays) Molecular markers of inflammation (gene expression, ELISA) Microscopy‐based ciliary dynamics & tissue integrity assessment	Provides a controlled preclinical ex vivo platform for the evaluation of candidate antimicrobials, antivirals, antifungals, or immunomodulatory agents in infected lung tissue prior to *in vivo* studies; cost‐effective and ethically favorable
**Toxicology**	Dose‐dependent tissue damage scoring (histology) Cytotoxicity & cell viability (resazurin assays, LDH release assays) Inflammatory gene expression Oxidative stress markers	Enables dose‐response assessment of environmental pollutants, inhaled compounds, or other xenobiotics in physiologically relevant lung tissue without live‐animal exposure (Eymieux et al., 2024)
**Experimental efficiency & 3Rs contribution**	Multiple replicates and conditions from one donor lung Parallel testing of pathogen strains, treatments, time points, and conditions with matched controls Abattoir‐sourced tissue (food production by‐product)	Reduces biological (inter‐animal) variability between experimental conditions Maximizes scientific output from a single tissue source Supports replacement and reduction principles (3Rs) Species applicability: murine, human, porcine, bovine, ferret, ovine, equine, canine lung tissue Comparative respiratory biology across species and zoonotic disease studies (Eymieux et al., 2024; Koziol‐White et al., 2024)

Here, we present a comprehensive, reproducible set of protocols for generating, culturing, and maintaining b‐PCLS, along with longitudinal viability assessment, as described in Basic Protocol 1. Basic Protocol 2 outlines the workflow for inoculating b‐PCLS with *Pasteurella multocida* (PM), a major bacterial contributor to BRD, enabling investigation of bacterial adherence and tissue‐level host–pathogen interactions. Basic Protocol 3 describes the experimental procedure for viral inoculation with bovine coronavirus (BCoV). Basic Protocol 4 details the extraction of RNA from culture supernatants and b‐PCLS. RNA isolated from culture supernatants enables monitoring of viral replication by RT‐qPCR, while an optimized workflow for RNA isolation from b‐PCLS yields high‐quality RNA that supports downstream transcriptomic analyses. This includes RNA‐seq‐based profiling of both host immune responses and Gram‐negative bacterial gene expression; the results will be reported separately. Together, these protocols establish b‐PCLS as a robust *ex vivo* platform for investigating bacterial adherence, viral replication, and the molecular mechanisms underlying tissue‐level host‐pathogen interactions relevant to BRD, while reducing reliance on live‐animal experimentation.

*NOTE*: All solutions, consumables, and equipment that come into contact with tissue must be sterile, and proper sterile technique should be used.

*NOTE*: All culture incubations are performed at 37°C and 5% CO_2_ unless otherwise specified.

*NOTE*: Common materials, reagents, and equipment are listed in Basic Protocol 1. To avoid repetition, subsequent protocols include only additional or protocol‐specific items not previously listed.

*NOTE*: All protocols involving animals must be reviewed and approved by the appropriate Animal Care and Use Committee and must follow regulations for the care and use of laboratory animals.

## Basic Protocol 1 GENERATION, MAINTENANCE, AND VIABILITY ASSESSMENT OF B‐PCLS

This protocol describes the preparation, culture, and viability assessment of bovine precision‐cut lung slices (b‐PCLS) from lung tissue obtained from freshly slaughtered cattle at a local abattoir. With minor modifications, the procedure can be adapted for lungs from other mammalian species.

### Materials

**Figure 1:**
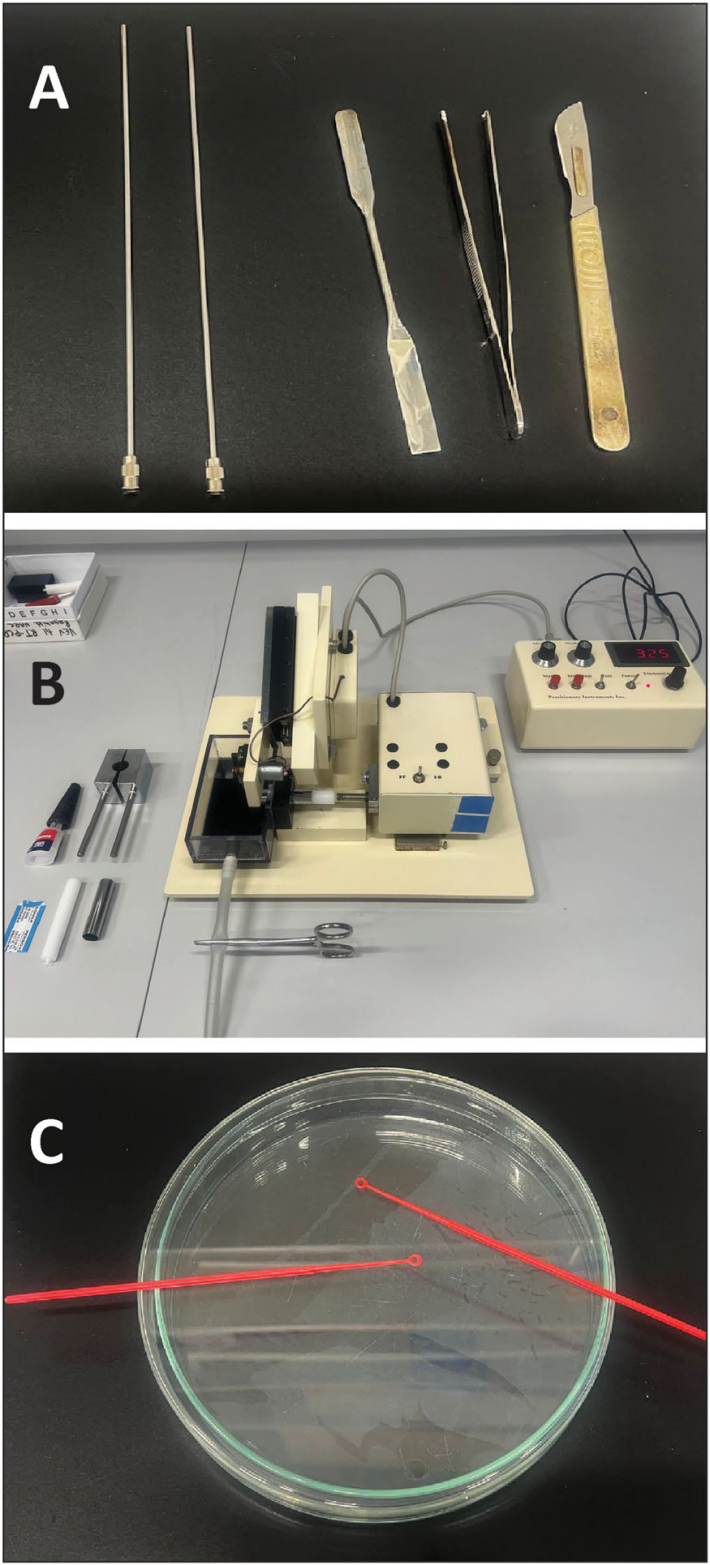
Representative equipment required for the generation of bovine precision‐cut lung slices (b‐PCLS), including (A) 150‐mm needles, spatula, tweezers, and scalpel; (B) vibrating microtome (Compresstome® VF‐300‐0Z, Precisionary Instruments) and specimen tube kit; and (C) large glass Petri dish with 5‐µL inoculation loops.

At the slaughterhouse
  Cryo‐foam transport box containing ice
  Cutting board
  Sharp knife
  75% (*v/v*) ethanol
  Disposable gloves
  Mini autoclave plastic bags for the lung lobes
In the laboratory
Reagents
  RPMI 1640 powder with L‐glutamine, without sodium bicarbonate (Biowest, cat. no. P0860‐N1L)
  RPMI 1640 medium with L‐glutamine, without HEPES (Thermo Fisher Scientific, cat. no. 12004997)
  Low‐melting‐point (LMP) agarose (Apollo Scientific, cat. no. BIA1189‐100G)
  Dulbecco's phosphate‐buffered saline (DPBS), without Ca²⁺ and Mg²⁺ (Thermo Fisher Scientific, cat. no. 14190250)
  PrestoBlue high‐sensitivity cell viability reagent (Thermo Fisher Scientific, cat. no. P50200)
  Triton X‐100 (Thermo Fisher Scientific, cat. no. A16046.AE)
  75% (*v/v*) ethanol
  25% (*v/v*) glycerol
Antibiotics for RPMI (RPMI‐AB)
  Penicillin‐Streptomycin (10,000 U/mL; Thermo Fisher Scientific, cat. no. 15140122)
  Gentamicin (10 mg/mL; Thermo Fisher Scientific, cat. no. 15710049)
  Amphotericin B (250 µg/mL; Sigma‐Aldrich, cat. no. A2942‐100ML)
  Kanamycin sulfate hydrate (PanReac AppliChem, cat. no. A1493.0005)
  Clotrimazole (Sigma‐Aldrich, cat. no. PHR1058‐1G)
Equipment
Biological safety cabinet
Water bath (37°C and 42°C)
Microwave oven
CO_2_ incubator set to 37°C and 5% CO_2_
Flat‐tip tweezers (Fig. 1A)
Scalpel (Fig. 1A)
Thin curved spatula or microspoon (Fig. 1A)
Forceps
Industrial blunt‐tip stainless‐steel needle, Luer lock, 14 G, 150 mm (Fig. 1A)
Vibrating microtome (Compresstome® VF‐300‐0Z, Precisionary Instruments) (Fig. 1B)
Specimen tube kit (Precisionary Instruments, cat. no. SKU VF‐BHSK‐VM‐15.5‐NC) (Fig. 1B)
Blades (Personna, cat. no. 74‐0002‐CTN) (Fig. 1B)
Superglue (Loctite) (Fig. 1B)
50‐mL syringes (Luer slip)
Cutting board and knife
Biopsy Punch, 8‐mm diameter (sterile)
Vacuum aspirator (Vacusafe aspiration system, Integra Biosciences)
Inverted light microscope (Invitrogen EVOS Microscope M5000 Imaging System)
Microplate reader (Tecan Spark multimode plate reader)
Glass bottles (100, 500, and 1,000 mL; Simax)
Glass beakers (250 mL, 400 mL, and 1,000 mL)
Glass Petri dishes, large size (Fig. 1C)
Glass pipettes (10 mL, 25 mL)
Glass Pasteur pipettes, 230 mm long (sterile)
Magnetic stirrer and stir bars
Horizontal shaker
Disposable sterile inoculating loops (5 µL) (Fig. 1C)
24‐well cell culture plates (Sarstedt, cat. no. NC0984607)
96‐well plates (Sarstedt, cat. no. NC9603080)
1000‐µL pipette and pipette tips
Cryo‐foam box containing ice

#### Procedure

The preparation and experimental use of bovine precision‐cut lung slices (b‐PCLS) are typically completed over multiple days. The duration may vary depending on the number of slices generated and the study design. A representative timeline is outlined below, with detailed timing for individual steps indicated within each protocol section.

**Day 1**: Lung collection, agarose inflation, vibratome slicing, selection of intact b‐PCLS, transfer to 24‐well plates, washing with DPBS, and overnight recovery incubation
**Day 2**: Assessment of ciliary activity and selection of viable b‐PCLS; evaluation of metabolic activity in representative slices, if required.
**Day 3 onward**: Initiation and progression of the bacterial or viral infection experiment according to the study design. RNA extraction and other endpoint analyses are performed after the infection period is complete; the timing may vary depending on the study design.

#### Preparation of reagents and equipment in the laboratory

##### Timing: ∼2 hr

Prepare all reagents and equipment before visiting the slaughterhouse. These steps may be completed one day in advance. If so, store RPMI and low‐melting‐point (LMP) agarose solutions at 4°C. On the day of use, remelt the agarose and equilibrate the agarose and RPMI in water baths set to 42°C and 37°C, respectively.

1 Autoclave all heat‐resistant instruments, including glassware, forceps, tweezers, spatulas, needles, and magnetic stir bars, as required.
2 Split a microtome blade longitudinally into two halves.
3 Attach one half of the blade to the vibratome's blade holder using superglue.
4 Mount the blade holder in the vibratome.
5 Decontaminate the vibratome chamber and blade with 75% ethanol.
6 Rinse thoroughly with DPBS and cover with aluminum foil until use.
7 Dissolve one sachet of RPMI powder in 500 mL autoclaved double‐distilled water to prepare 2× RPMI (for later dilution to 1×).
  Use a magnetic stirrer to ensure complete dissolution.
8 Equilibrate the 2× RPMI in a 37°C water bath.
9 Prepare two 500‐mL bottles, each containing 250 mL of 3% (*w/v*) LMP agarose in autoclaved double‐distilled water.
10 Prepare one 100‐mL bottle containing 50 mL of 4% (*w/v*) LMP agarose in autoclaved double‐distilled water.
11 Melt agarose solutions in a microwave using short heating cycles until fully dissolved and visually clear.
12 Keep melted agarose solutions in a 42°C water bath until use.

#### Preparation of antibiotic‐supplemented RPMI (RPMI‐AB)

##### Timing: 15 min

13 Supplement one commercial RPMI solution bottle (500 mL) with antibiotics to the final concentrations listed in Table 2. Mix gently to ensure complete dissolution. Store the prepared medium at 4°C until use.
  Three RPMI solutions are used in these protocols:
  2× RPMI solution made from RPMI powder, used for lung inflation after being mixed with 3% LMP agarose.
  Commercial RPMI solution without supplementation, used for metabolic assessment and infection experiments.
  Commercial RPMI solution + antibiotics (RPMI‐AB), used for maintenance culture of slices.

**Table 2:** Antibiotics added to the RPMI culture medium for bovine precision‐cut lung slices (b‐PCLS)

Antibiotic	Stock concentration per mL	Final concentration per mL	Volume (mL) to add per 500 mL RPMI	Notes
**Penicillin‐Streptomycin**	10,000 U penicillin 10 mg streptomycin	100 U 100 µg	5	Store at −20°C
**Gentamicin**	10 mg	50 µg	2.5	Store at 4°C
**Amphotericin B**	250 µg	2.5 µg	5	Store at −20°C
**Kanamycin**	25 mg	50 µg	1	Dissolve in 25% (*v/v*) glycerol, mix, filter using a 0.22‐µm filter, and store at −20°C
**Clotrimazole**	1 mg	1 µg	0.5	Dissolve in 75% (*v/v*) ethanol, mix, filter using a 0.22‐µm filter, and store at −20°C

#### Lung tissue collection at the slaughterhouse (Fig. 2A)

##### Timing: 30–60 min

14 From clinically healthy cattle (this protocol; 2–3 years old): Collect lungs immediately after slaughter.
15 Place lungs on a cutting board.
16 Select the accessory and cranial lung lobes and excise them completely at their bases using a knife.
17 Exclude lobes showing visible pathological lesions (e.g., consolidation, hemorrhage, abscesses) or mechanical damage resulting from slaughter or handling (e.g., deep incisions, lacerations).
18 Transfer the selected lobes into mini autoclave plastic bags and place them on ice.
19 Transport the lung lobes to the laboratory for immediate processing (within 1 hr).

**Figure 2:**
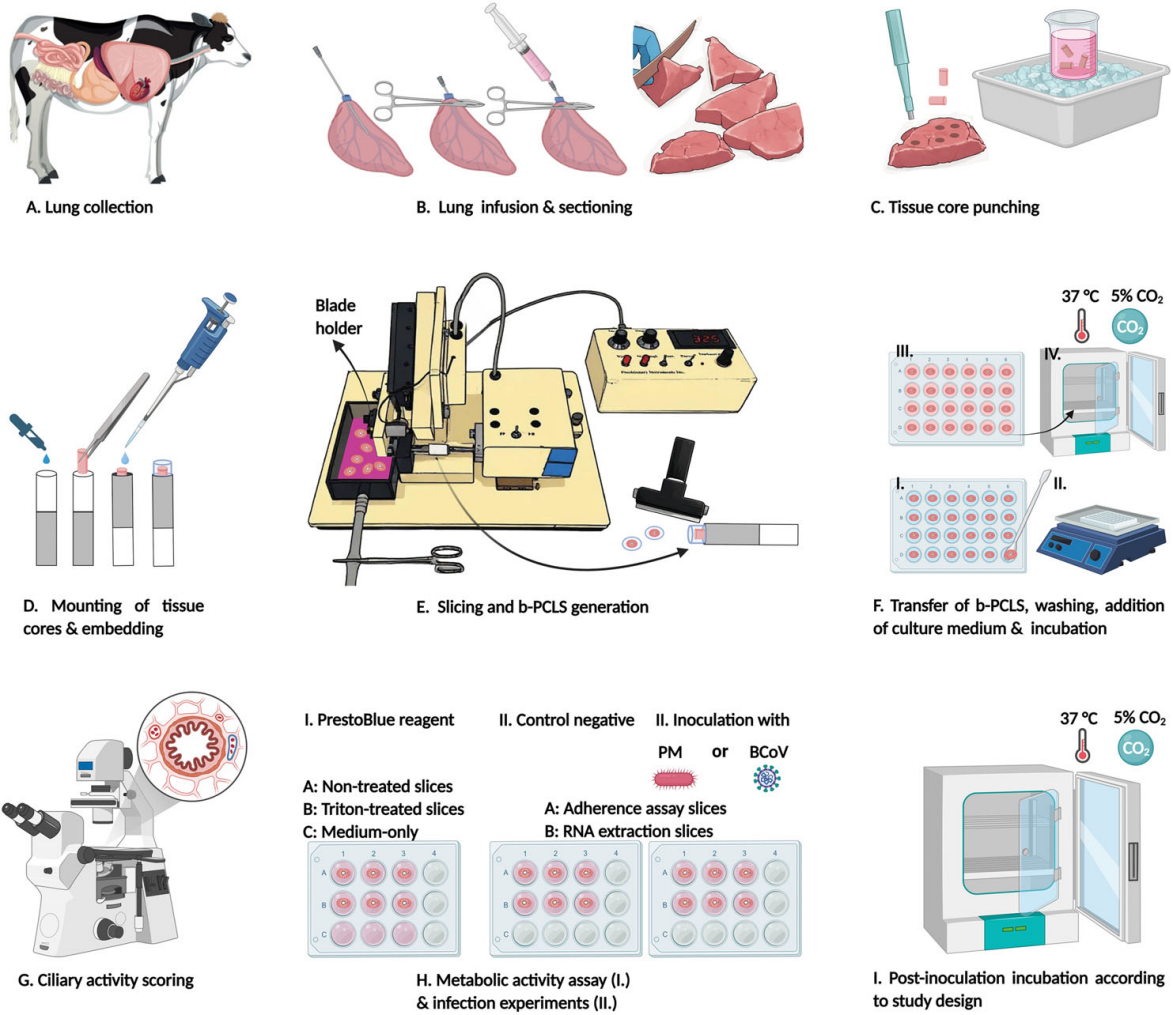
Schematic overview of Basic Protocols 1–3 illustrating (A‐F) preparation and culture of bovine precision‐cut lung slices (b‐PCLS); (G) assessment of tissue viability by ciliary activity scoring; (H (I)) assessment of tissue viability by metabolic activity assay with PrestoBlue HS Cell Viability Reagent; (H (II)) inoculation with *Pasteurella multocida* (PM) or bovine coronavirus (BCoV) with subsequent incubation periods (I).

#### Lung lobe inflation with agarose (Fig. 2B and Fig. 3A–B)

##### Timing: 1 hr

Before lung infusion, mix each bottle of 3% (*w/v*) LMP agarose (250 mL) with 250 mL of 2× RPMI to obtain a final solution of 1.5% agarose in 1× RPMI. Maintain this mixture and the 4% (*w/v*) LMP agarose at 42°C until use.

**Figure 3:**
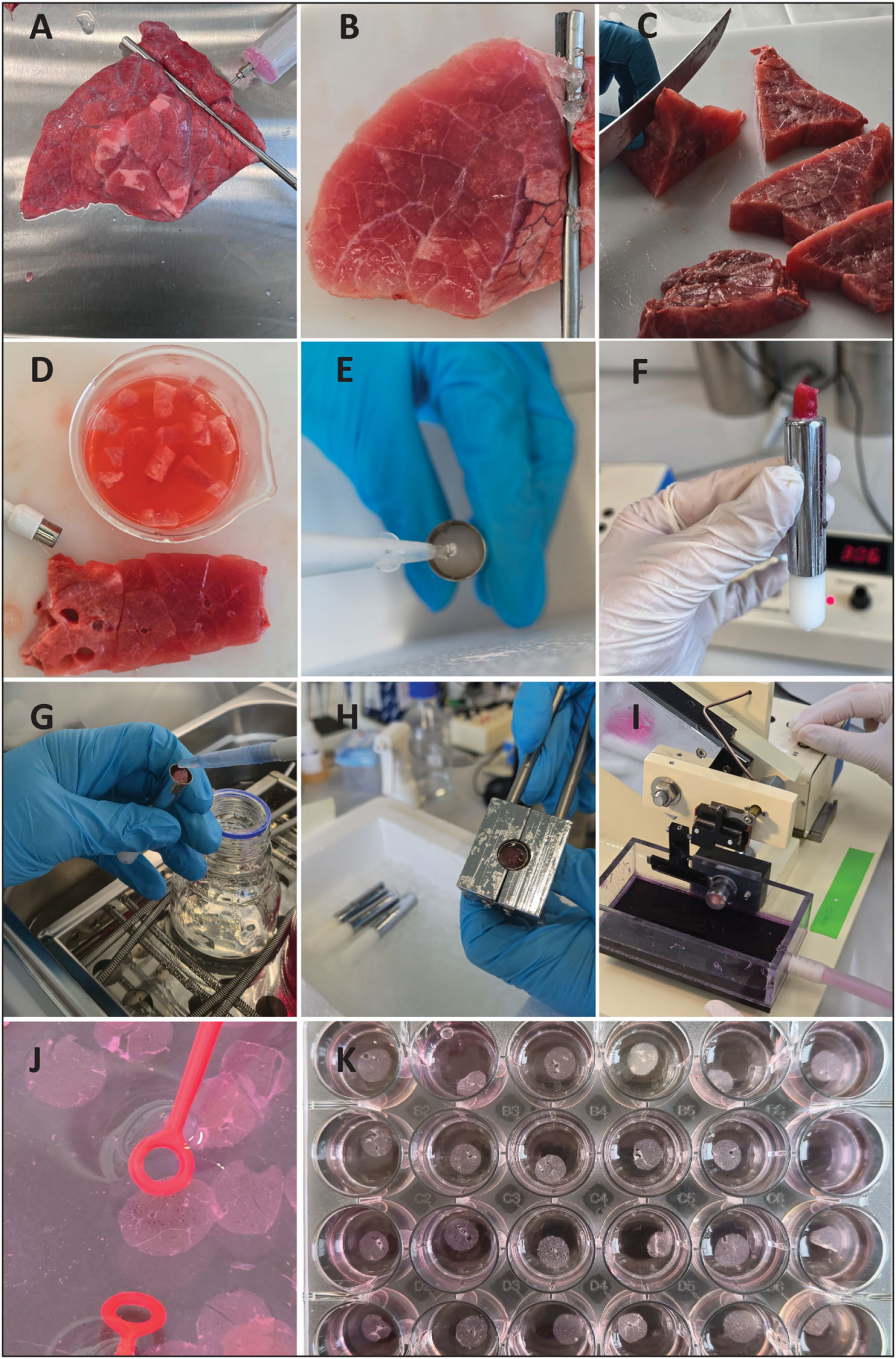
Representative workflow for the generation of bovine precision‐cut lung slices (b‐PCLS). Selected steps from tissue preparation to culture are shown: (A) infusion of a lung lobe with RPMI 1640–low melting point (LMP) agarose mixture; (B) enlarged lung lobe after agarose solidification on ice; (C) lung lobe sectioning; (D) punching of tissue cores; (E) application of superglue onto the surface of a specimen tube; (F) mounted tissue core onto the specimen tube; (G) embedding of the tissue core in 4% LMP agarose; (H) solidification of the embedded tissue using a chilled block; (I) placement of the specimen tube with embedded tissue in the vibratome for slicing at a 90° angle to the tissue core; (J) generated b‐PCLS; (K) transferred b‐PCLS into a 24‐well plate containing RPMI supplemented with antibiotics (RPMI‐AB) for culture.

20 Disinfect the laboratory bench and cutting board.
21 Place the lung lobes on the cutting board.
22 Identify the main (largest) bronchus in each lung lobe.
23 Carefully insert a blunt‐tip needle into the bronchus.
24 Gently advance the needle along the airway without applying force.
25 Secure the needle in place with forceps.
26 Load a 50‐mL syringe with the agarose‐RPMI mixture.
27 Slowly infuse the lung lobe while gradually withdrawing the needle to promote homogeneous agarose distribution throughout the tissue (Fig. 2B and Fig. 3A).
28 Continue infusion until the lobe is uniformly expanded and firm to gentle palpation (Fig. 3B).
  Avoid excessive infusion pressure to prevent bronchiolar rupture.
29 Place the infused lobes on ice for 30 min to allow agarose to solidify.

#### Sectioning and vibratome slicing (Fig. 2B–E and Fig. 3C–J)

##### Timing: ∼3 hr

This timing corresponds to the preparation of approximately 72 suitable b‐PCLS (three 24‐well plates).

30 Remove forceps and needles and transfer the agarose‐inflated lung lobes to a newly disinfected cutting board.
31 Section each lung lobe into transverse slices approximately 2 cm thick (Fig. 2B and Fig. 3C).
  Sections should be cut perpendicular to the main bronchial axis to obtain bronchioles in cross‐section (circular profiles) rather than longitudinally (oval or elongated profiles).
32 Using an 8‐mm biopsy punch, excise cylindrical tissue cores from the lung sections (Fig. 2C and Fig. 3D).
  Position the biopsy punch to select tissue cores containing centrally located small bronchioles; avoid large conducting airways.
33 Submerge tissue cores in cold RPMI and keep on ice until mounting (Fig. 2C and Fig. 3D).
34 Apply a small drop of superglue to the surface of the specimen tube (Fig. 2D and Fig. 3E).
35 Using blunt‐tip tweezers, remove one tissue core at a time and gently blot excess medium on absorbent paper.
36 Mount the tissue core onto the specimen tube at the site of the glue (Fig. 2D and Fig. 3F).
  Residual glue on the specimen tube can interfere with mounting subsequent tissue cores. Use a scalpel to gently clean the tube surface between mounts.
37 Gently raise the surrounding wall of the specimen tube to stabilize the tissue.
38 Surround the tissue core with 600–800 µL of molten 4% LMP agarose (Fig. 2D and Fig. 3G).
39 Allow the agarose to solidify using a chilled block (Fig. 3H).
  Ensure the tissue core is completely embedded in 4% LMP agarose to provide structural support during slicing.
40 Place the specimen tube into the slot within the vibratome chamber.
41 Advance the specimen tube until it reaches the stopper.
42 Adjust the blade holder to a 90° angle relative to the tissue core (Fig. 2E and Fig. 3I).
43 Fill the vibratome chamber with cold RPMI.
44 Press the forward button on the motor drive until the bar contacts the back of the specimen tube (Fig. 2E).
  This ensures the specimen tube advances incrementally with each blade pass.
45 Set the slicing parameters: advance speed (9), oscillation amplitude (9), and slice thickness (300 µm), and start slicing (Video 1).

**Video 1:** Slicing procedure for the generation of bovine precision‐cut lung slices (b‐PCLS) using a vibrating microtome (Compresstome® VF‐300‐0Z, Precisionary Instruments). The video shows positioning of the agarose‐embedded tissue core, slicing with the blade, and the representative b‐PCLS obtained.

46 Collect b‐PCLS gently using a spatula, microspoon, or loop (Fig. 3J).
47 Immediately transfer the b‐PCLS into a glass bottle containing 50 mL prewarmed RPMI‐AB (37°C).
  During slicing, the collection bottle may be placed in a 37°C water bath adjacent to the vibratome to maintain physiological temperature until all slices are collected.
  The cranial and accessory lung lobes are the smallest and easiest to inflate and handle. Typically, 15–20 tissue cores containing centrally located bronchioles can be obtained per lobe, with each tissue core yielding up to ∼50 b‐PCLS of 300 µm thickness. However, due to the lobular architecture and bronchiolar branching pattern of bovine lung tissue, not all generated slices are intact or contain a centrally located bronchiole. Some slices may display branched, partial, or off‐center bronchioles, or reduced ciliary activity, rendering them unsuitable for downstream analyses. It is therefore recommended to generate an excess of b‐PCLS when designing the experiment and treatment groups.

#### Culture and maintenance of b‐PCLS (Fig. 2F and Fig. 3K)

##### Timing: ∼1.5 hr

48 Place the bottle containing b‐PCLS in a biological safety cabinet.
49 Pour the RPMI‐AB containing the b‐PCLS into a glass Petri dish.
50 Select intact b‐PCLS that have centrally located bronchioles.
  Placing the Petri dish on a black background enhances contrast and facilitates visualization of bronchiolar cross‐sections during slice selection.
51 Transfer the selected b‐PCLS individually using a sterile spatula or loop into the wells of three 24‐well plates, each prefilled with 1 mL prewarmed (37°C) DPBS (Fig. 2F, I).
52 Wash slices three times with 1 mL prewarmed DPBS to remove residual agarose from the bronchioles (Fig. 2F, I and Fig. 2F, II).
  All washing steps of slices include 5‐min agitation intervals on a horizontal shaker at 100 rpm.
53 After the final wash, add 1 mL prewarmed RPMI‐AB per well (Fig. 2F, III and Fig. 3K).
54 Incubate overnight at 37°C and 5% CO_2_ (Fig. 2F, IV).
  For longer‐term culture, replace the medium with RPMI without antibiotics the following day (after recovery from slicing), and replace the medium every other day.

#### Assessment of b‐PCLS viability

The viability of b‐PCLS can be assessed using two complementary approaches:

A. Ciliary activity
B. Metabolic activity using the PrestoBlue high‐sensitivity cell viability reagent (PB)

#### A. Assessment of ciliary activity (Fig. 2G and Fig. 4)

##### Timing: 1–2 min per slice

**Figure 4:**
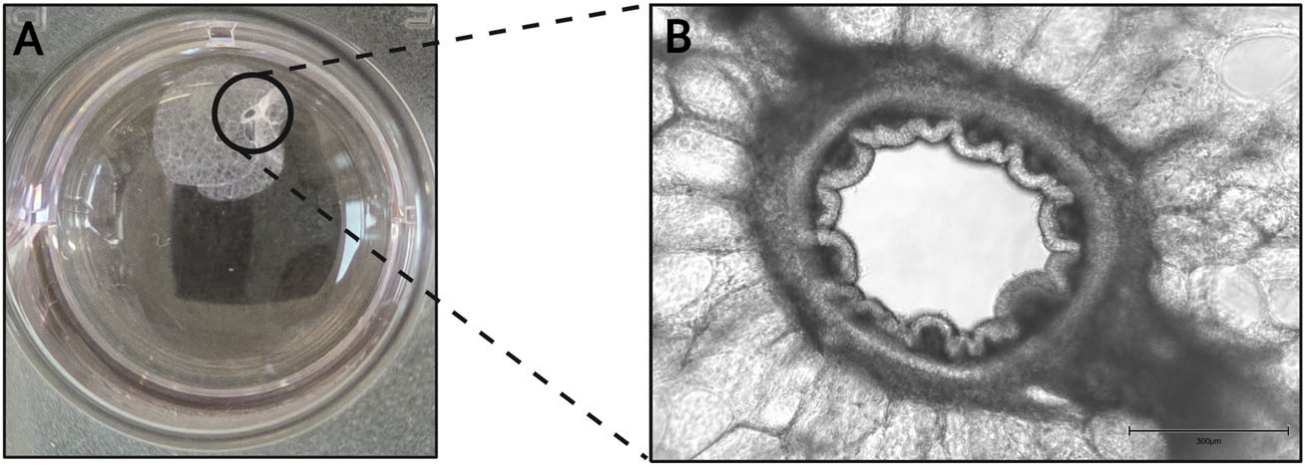
Visualization of a bronchiole within a bovine precision‐cut lung slice (b‐PCLS). (A) Macroscopic view of a single b‐PCLS showing a visible bronchiole. (B) Higher‐magnification view of the bronchiole under an inverted microscope (10×). Scale bar: 300 µm

55 The day after slicing, remove one 24‐well plate at a time from the incubator.
56 Examine each well under an inverted light microscope at 10–20× magnification, focusing on the bronchioles (Fig. 4A–B).
57 Observe the ciliary beating along the visible lining of the bronchiolar lumen (Fig. 4 and Video 2).

**Video 2:** Ciliary activity of a bronchiole in a bovine precision‐cut lung slice (b‐PCLS) visualized under an inverted microscope at 20× magnification.

58 Estimate the proportion of the ciliated epithelial lining that exhibits active ciliary beating.
59 Express this ciliary activity as a percentage of the total visible bronchiolar lining (e.g., 100% when cilia beat along the entire bronchiole; ∼80% when activity is observed along approximately four‐fifths of the lining).
  Where possible, use b‐PCLS displaying ≥80% ciliary activity for downstream experiments.

#### B. Assessment of metabolic activity with PrestoBlue (PB) reagent (Fig. 2H, I)

##### Timing: ∼2.5 hr

From the pool of b‐PCLS that pass the ciliary activity threshold (≥80%), select at least six slices for metabolic activity assessment. Allocate three slices to the untreated experimental group and three slices to a Triton X‐100–treated group to induce cellular lysis, serving as a no‐metabolic‐activity control.

60 Allow PB to reach room temperature (RT).
61 Prepare a 10% (*v/v*) PB working solution in prewarmed RPMI (37°C), in a volume sufficient for all samples and controls (500 µL per well).
62 Transfer the six selected b‐PCLS into a new 24‐well (or 12‐well) plate prefilled with 500 µL prewarmed RPMI per well using a 5‐µL loop.
63 For “no‐metabolic‐activity controls”, treat the slices with Triton X‐100 to a final concentration of 10% (*v/v)*.
64 Incubate the plate for 1 hr at 37°C and 5% CO_2_.
65 Remove the culture medium from all wells using a vacuum aspirator.
66 Add 500 µL PB working solution to each well containing a b‐PCLS, including no‐metabolic‐activity controls.
67 Include three wells containing 500 µL PB working solution only (no b‐PCLS) as medium background controls.
68 Gently tilt the plate to ensure uniform coverage of the slices.
69 Incubate for 1 hr at 37°C and 5% CO_2_.
70 Transfer 150 µL of supernatant from each well to a 96‐well plate for fluorescence measurements.
71 Measure fluorescence intensity using a microplate reader with the following settings:
  Excitation: 535 nm (bandwidth 25 nm)
  Emission: 595 nm (bandwidth 35 nm)
  Absorbance: 570 nm with reference at 600 nm
    Correct fluorescence values of untreated and Triton‐treated slices by subtracting the mean signal of the medium background control wells to account for the baseline fluorescence of the PB working solution prepared in RPMI.
72 After measurement, remove the PB solution from the 24‐well plate using a vacuum aspirator.
73 Wash b‐PCLS once with 1 mL DPBS.
74 Replace with 1 mL fresh RPMI.
75 Return the plate to the incubator at 37°C and 5% CO_2_.
  Metabolic activity assessment is performed on representative slices and is not a selection criterion for experimental slices.
  PB is non‐toxic, and the assay can be repeated at defined time points (e.g., days 1, 3, 5, and 7) on the same slices to monitor longitudinal viability.

## Basic Protocol 2 INOCULATION OF B‐PCLS WITH *PASTEURELLA MULTOCIDA* (Fig. 2H, II, and 2I)

This protocol describes the preparation and culture of *P. multocida* and the inoculation of viable b‐PCLS with this bacterium as an *ex vivo* infection model for respiratory pathogens. This bacterial isolate originated from a BRD case in a study on Norwegian herds (Ahmadi et al., 2026; Ånestad et al., 2026).

All tools, reagents, and work surfaces should be sterile, and procedures should be carried out aseptically.

All work involving infection experiments must be performed under biosafety level 2 (BSL‐2) conditions in accordance with institutional and national biosafety regulations.

### Materials

Viable b‐PCLS
Bacterial culture
*P. multocida* strain (Subspecies *multocida*, Capsular type A, LPS group 3, sequence type ST79, isolated from a Norwegian calf with BRD).
  Blood agar plates
  Brain Heart Infusion (BHI) broth
Reagents
RNAlater™ Stabilization Solution (Thermo Fisher Scientific, cat. no. AM7021)
Antibiotic‐free RPMI 1640
DPBS
Equipment
  Shaking incubator (37°C, 200 rpm)
  Spectrophotometer (Genesys 20)
  Centrifuge (4°C, 12,000 rpm)
  50‐mL Falcon tubes
  100‐mL Erlenmeyer flask
  12‐well plates (Sarstedt, cat. no. NC1408617)
  1.5‐mL microtubes
  100 µL pipettes and filter tips
  FastPrep™‐24 or FastPrep™‐96 instrument (MP Biomedicals)
  2‐mL Lysing Matrix D tubes, 1.4‐mm zirconium‐silicate beads (MP Biomedicals, cat. no. MP116913100)

#### Procedure

The bacterial culture workflow is coordinated with lung collection and b‐PCLS preparation. The following schedule outlines *P. multocida* culture preparation:

**Day 1 (one day before lung collection)**: Initial plating of *P. multocida* from a glycerol stock onto blood agar.
**Day 2 (day of lung collection and b‐PCLS generation)**: Streak a single, well‐isolated colony onto fresh blood agar to obtain a clonal working culture.
**Day 3 (day of ciliary activity assessment)**: Preparation of the bacterial preculture in BHI broth.
**Day 4 (day of inoculation; two days after b‐PCLS generation)**: Preparation of the bacterial inoculum (mid‐logarithmic‐phase culture and inoculum dose adjustment), followed by inoculation of b‐PCLS.

#### Preparation of *P. multocida* clonal working culture

##### Timing: 2 days

1 Thaw a *P. multocida* (PM) glycerol stock (−80°C) briefly on ice.
2 Streak PM onto a blood agar plate using standard quadrant streaking.
3 Incubate the plate overnight at 37°C with 5% CO_2_.
4 The following day, select a single well‐isolated colony and re‐streak it onto a fresh blood agar plate.
5 Incubate overnight at 37°C with 5% CO_2_ to obtain a fresh clonal culture.

#### Preparation of *P. multocida* preculture and inoculum

##### Timing: ∼20 hr

6 Using an inoculation loop, pick material from 2 to 3 freshly grown, well‐isolated colonies of PM.
7 Inoculate into 20 mL of BHI broth in a 50‐mL Falcon tube (preculture).
8 Incubate overnight at 37°C without agitation, placing the preculture tube on ice inside the incubator.
  Overnight cultures serve as precultures and should not be used directly for experimental inoculation. Placing the tube on ice inside the incubator reduces the effective culture temperature, thereby limiting excessive bacterial replication and preventing transition into the late logarithmic or stationary phase while maintaining bacterial viability.
9 The following day, measure the optical density of the preculture at 600 nm (OD₆₀₀).
10 Inoculate an aliquot of the preculture into 30 mL fresh BHI broth in an Erlenmeyer flask to achieve an initial OD₆₀₀ of 0.05.
11 Incubate at 37°C with shaking at 200 rpm for ∼3 hr to reach the mid‐logarithmic growth phase.
12 Measure OD₆₀₀ and estimate the bacterial concentration (colony‐forming units, CFU) using a previously established OD–CFU calibration curve.
  The OD–CFU relationships must be determined empirically for each bacterial strain, typically by generating a standard growth curve through plating serial dilutions at defined OD values.
13 Pellet bacteria by centrifugation at 12,000 × g and 4°C for 10 min.
14 Discard the supernatant and resuspend the bacterial pellet in antibiotic‐ and serum‐free RPMI to the original culture volume.
  Lower centrifugal forces may be used; however, this can result in less compact pellets and loss of bacteria. Some PM strains form more mucoid or poorly compacting pellets, so removing the supernatant may reduce bacterial recovery. Adjusting the resuspension volume in RPMI or normalizing based on OD₆₀₀ may be required to achieve the desired inoculation dose.
15 Prepare serial dilutions in RPMI to obtain the target concentration (10⁶ CFU/mL in this experiment) in a volume sufficient for all b‐PCLS treatment groups.
16 Plate the appropriate dilutions (e.g., 10^−3^ and 10^−4^ for the target dose of 10^6^ CFU/mL) on blood agar to confirm the inoculation dose.

#### Inoculation of b‐PCLS with *P. multocida*

##### Timing: 3 hr, variable depending on pathogen and study design

Infection experiments are best performed within the first 3 to 4 days after b‐PCLS generation, when slices have recovered and ciliary activity has been assessed. Preparation of the bacterial inoculum should be coordinated to ensure the availability of fresh, mid‐logarithmic‐phase bacteria at the time of b‐PCLS inoculation.

17 Transfer one b‐PCLS per well into a 12‐well plate containing 1 mL prewarmed DPBS.
18 Wash b‐PCLS twice with 1 mL DPBS using gentle agitation for 5 min per wash to remove residual antibiotics.
19 Aspirate DPBS after the final wash.
20 Add 1 mL bacterial suspension (10⁶ CFU/slice, prepared in antibiotic‐free RPMI) to each well.
21 Include uninfected control wells containing b‐PCLS incubated in antibiotic‐free RPMI only.
22 Incubate plates at 37°C and 5% CO_2_ for the desired time points according to the experimental design (e.g., 30, 60, 120 min, and 3 hr post‐inoculation).
23 After each defined time point, remove the corresponding plate from the incubator for downstream processing.
  Organize b‐PCLS in 12‐well plates such that each plate corresponds to a single incubation time point. This prevents disturbance of slices designated for later time points. For each time point, keep uninfected control slices in a separate plate from infected slices to avoid cross‐contamination.

#### Post‐inoculation processing and downstream applications

##### Ciliary activity assessment

24 Examine b‐PCLS to estimate changes in ciliary activity compared with uninfected control slices at defined time points (up to 3 hr in this experiment; see Video 3).

**Video 3:** Ciliary activity of a bronchiole in an inoculated bovine precision‐cut lung slice (b‐PCLS) with *Pasteurella multocida*, 3 hr post‐inoculation, visualized under an inverted microscope at 40× magnification.

#### Bacterial adherence or invasion assays (Group A replicates, Fig. 2H, II)

25 Wash slices three times with 1 mL of DPBS, gently agitating for 5 min per wash, to remove non‐adherent bacteria.
26 Transfer slices into Lysing Matrix D tubes containing 1 mL DPBS.
27 Homogenize tissue using the FastPrep‐24 instrument (3 × 30 s at 5 m/s) with 1‐min intervals on ice between cycles.
28 Plate appropriate serial dilutions onto blood agar to quantify adherent and/or invasive bacteria (for CFU determination).

#### RNA preservation (Group B replicates, Fig. 2H, II)

29 Wash slices three times with an RNA stabilization reagent (e.g., RNAlater) using gentle agitation for 5 min per wash.
  Washing removes non‐adherent bacteria, while immediate exposure to RNAlater stabilizes both host and bacterial RNA, minimizing transcriptional changes during sample handling.
30 Transfer slices into microcentrifuge tubes containing 500 µL of RNAlater.
31 Store at −80°C until RNA extraction.

#### Additional downstream applications

Depending on the experimental design, additional analyses may include:

DNA extraction for quantification of bacterial genomes
Histopathological analyses (e.g., immunohistochemistry or immunofluorescence)
Cytotoxicity assays

## Basic Protocol 3 INOCULATION OF B‐PCLS WITH BOVINE CORONAVIRUS

This protocol details the inoculation of b‐PCLS with bovine coronavirus (BCoV) originating from a BRD case (nasal swabs) and adapted to the human rectal tumor cell line HRT‐18G (up to passage 14) (Shakya et al., 2022, 2023). The virus stock has a titer of 2.15 × 10^6^ TCID_50/mL_. The focus is on preparing the viral inoculum and inoculating slices for downstream analyses.

BCoV is a Biosafety Level 2 (BSL‐2) pathogen. Follow all appropriate guidelines and regulations for the use and handling of pathogenic microorganisms.

### Materials

Viable b‐PCLS
Fetal bovine serum (Thermo Fisher Scientific, cat. no. 10270106)
Inoculation medium (antibiotic‐ and serum‐free RPMI 1640)
Maintenance medium (RPMI 1640 supplemented with 1% heat‐inactivated fetal bovine serum (FBS) and antibiotics as listed in Table 2 in Basic Protocol 1)

#### Inoculation of b‐PCLS with BCoV (Fig. 2H, II, and 2I)

##### Timing: 48 hr in this experiment (variable depending on the virus and study design)

1 Prepare the viral inoculum by diluting the BCoV stock 1:10 in RPMI medium to achieve a final concentration of 2.15 × 10^5^ TCID_50/mL_.
2 Transfer one b‐PCLS per well into 12‐well plates containing 1 mL of prewarmed DPBS.
3 Wash slices twice with 1 mL of DPBS, using 5‐min agitation intervals.
4 Aspirate DPBS and add 350 µL of diluted BCoV inoculum to each well designated for infection.
  The virus suspension volume should cover the b‐PCLS surface without fully submerging the slice, ensuring effective viral contact.
5 Incubate the plate for 2 hr at 37°C and 5% CO_2_ to allow viral attachment to epithelial cells (adsorption period).
6 Wash inoculated slices twice with 1 mL DPBS, using 5‐min agitation intervals to remove unbound virus.
7 Add 1 mL of maintenance medium (RPMI‐FBS‐AB) to each well.
8 Incubate plates at 37°C and 5% CO_2_ for the desired duration of the infection experiment.
  For the early (∼2 hr) time point, proceed with sampling after a short incubation period (≤30 min) following the addition of maintenance medium (see next section, step 10).

#### Sampling at defined time points

Two distinct subsets of b‐PCLS are used.

**Subset 1**: Maintained for longitudinal monitoring. Small aliquots of supernatant (50 µL) are collected from the same slices at defined time points (e.g., 2 and 48 hr post‐inoculation) to assess viral replication over time by RT‐qPCR.
**Subset 2**: Harvested at defined time points for downstream transcriptomic analyses. For these slices, supernatants can also be collected immediately before harvesting. The slices are then preserved in RNAlater for tissue RNA extraction. Confirmation of viral replication in these samples by RT‐qPCR is recommended as a quality‐control step before proceeding with RNA extraction from b‐PCLS tissue.
  Supernatant collection follows the same procedure for both subsets and is described in this section. Viral RNA extraction from supernatants and RT‐qPCR analysis are detailed in Basic Protocol 4.

#### 2 hr post‐inoculation (early/baseline time point)

9 Remove the plate from the incubator after the adsorption period and after a short incubation following the addition of maintenance medium.
10 Collect 50 µL of each supernatant in 1.5‐mL microtubes and store at −80°C.
  For the longitudinal monitoring subset (Subset 1), return the slices to the incubator until the next time point.
11 For the subset intended for transcriptomics (Subset 2), after supernatant collection, wash the slices once with RNAlater.
12 Transfer the slices into microtubes containing 500 µL of RNAlater and store at −80°C for RNA extraction.

#### 48 hr post‐inoculation

13 Examine b‐PCLS to estimate ciliary activity compared to negative control slices.
14 Collect 50 µL of supernatant into microtubes and store at −80°C.
  Supernatants can be used for RT‐qPCR (evaluation of viral replication) or cytokine measurements.
15 Wash slices three times with RNAlater (5‐min agitation intervals).
16 Transfer slices into microtubes containing 500 µL RNAlater and store at −80°C for RNA extraction.
  Organize plates so that each time point is processed independently to avoid disturbing slices designated for later time‐point sampling. Separate non‐inoculated (negative control) plates from inoculated plates to prevent cross‐contamination.
  Additional downstream analyses may include metabolic activity, histopathology, immunostaining, or cytotoxicity measurements, depending on the experimental design.

## Basic Protocol 4 EXTRACTION of RNA FROM CULTURE SUPERNATANTS AND B‐PCLS

This protocol is divided into two sections:

4A. Viral RNA extraction from culture supernatants

4B. Total RNA extraction from b‐PCLS

Viral RNA extracted from culture supernatants is used to document viral replication following inoculation. Total RNA extracted from b‐PCLS enables downstream applications, such as host transcriptomic analyses and, where applicable, assessment of tissue‐associated bacterial gene expression.

### Materials

RNase AWAY™ Surface Decontaminant (Thermo Fisher Scientific, cat. no. 7002PK)
RNAlater (Thermo Fisher Scientific, cat. no. AM7021)
RNeasy Lysis Buffer (Buffer RLT, Qiagen, cat. no. 79216)
β‐Mercaptoethanol (β‐ME) (Sigma‐Aldrich, cat. no. M6250‐100ML)
2‐Propanol (Sigma‐Aldrich, cat. no. I9516‐25ML)
ROTI® Aqua‐Phenol/Chloroform/Isoamyl alcohol (25:24:1), pH 4.5–5 (Carl Roth, cat. no. X985.1)
Chloroform–isoamyl alcohol mixture, 49:1 (Sigma‐Aldrich, cat. no. 25668‐100ML)
Proteinase K solution, 600 mAU/mL (Qiagen, cat. no. 19131)
NaCl, 5 M, RNase‐free (Thermo Fisher Scientific, cat. no. AM9759)
UltraPure™ Glycogen (Thermo Fisher Scientific, cat. no. 10814010)
Nuclease‐free water
MagMAX™ *mir*Vana™ Total RNA Isolation Kit (Thermo Fisher Scientific, cat. no. A27828)
TaqMan™ Fast Virus 1‐Step Master Mix for qPCR (Thermo Fisher Scientific, cat. no. 4444432)
QIAamp Viral RNA Mini Kit (Qiagen, cat. no. 52906)
Qubit™ RNA Broad Range (BR) Assay kit (Invitrogen™, Thermo Fisher Scientific, cat. no. Q10211)
RNA ScreenTape Analysis (Agilent Technologies, cat. no. 5067‐5576)
Chemical fume hood
Thermomixer/horizontal shaker
FastPrep^®^‐96 high‐throughput bead‐beating grinder and lysis system (MP Biomedicals)
qPCR machine (Agilent AriaMx Real‐Time PCR system)
PCR machine (iCycler)
NanoDrop microvolume spectrophotometer (mySPEC)
Fragment analyzer (Agilent 4200 TapeStation)
TapeStation loading tips (Agilent Technologies, cat. no. NC1822523)
Qubit 4 Fluorometer (Thermo Fisher Scientific)
Qubit™ Assay Tubes (Thermo Fisher Scientific, cat. no. Q32856)
RNase‐free microcentrifuge tubes, low‐binding, 2 mL (Fisher Scientific, cat. no. 10318574)
RNase‐free microcentrifuge tubes, low‐binding, 1.5 mL (Fisher Scientific, cat. no. 10409044)
2‐mL BeadBug^™^ prefilled tubes, 0.1‐mm zirconium beads (Sigma‐Aldrich, cat. no. Z763764‐50EA)
2‐mL Lysing Matrix D tubes, 1.4‐mm zirconium‐silicate beads (MP Biomedicals, cat. no. MP116913100)
Magnetic stand

#### 4A – Viral RNA extraction from culture supernatants

##### Timing: ∼2 hr (∼4 hr, including RT**‐**qPCR run time)

1 Thaw collected culture supernatants from BCoV‐inoculated b‐PCLS at defined time points (e.g., 2 and 48 hr post‐inoculation) on ice.
2 Extract viral RNA using the QIAamp Viral RNA Mini Kit according to the manufacturer's instructions.
  The starting material volume is 50 µL and is adjusted to a final volume of 140 µL with DPBS prior to extraction, following the manufacturer's recommendations.
3 At the final step, elute RNA in 30 µL of AVE buffer.
4 Perform RT‐qPCR using TaqMan™ Fast Virus 1‐Step Master Mix.
5 Prepare reactions according to the manufacturer's protocol using BCoV‐specific primers and probe (Table 3) and the thermal cycling conditions in Table 4.

**Table 3:** Primers, probe, and reaction composition for RT‐qPCR detection of bovine coronavirus (BCoV) RNA

Component	Final concentration	Sequences 5′ to 3′	Volume per reaction (µL)
4× TaqMan™ Fast Virus 1‐Step Master Mix	1×	_	5
BCoV1F20 (forward primer)	400 nM	TGGTGTCTATATTCATTTCTGCTG	0.4
BCoV1R89 (reverse primer)	400 nM	GGCCACTGCCTAGGATACA	0.4
BCoV1P48 (probe)	800 nM	{FAM} ACACGTCCCTGGCTGAAAGCTG {BHQ1}	0.8
RNA template	_	_	2
Nuclease‐free water	_	_	11.4
Total volume	_	_	20

**Table 4:** Thermal cycling conditions for RT‐qPCR detection of bovine coronavirus (BCoV) RNA

Step		Temperature (°C)	Time	Cycles
**Reverse transcription**		50	5 min	1
**Initial denaturation**		95	20 s	1
**Amplification**	Denaturation	95	15 s	45
	Annealing/extension	58	1 min	45

#### 4B – Total RNA extraction from b‐PCLS (Fig. 5)

High‐quality RNA isolation from PCLS is challenging because agarose inflation can interfere with efficient RNA recovery, often resulting in low yield and reduced purity. This protocol describes the extraction of total RNA from uninfected, bacterially infected, or virally infected b‐PCLS. To overcome agarose‐associated limitations, the workflow integrates and modifies two previously published PCLS RNA extraction approaches (Langwiński et al., 2024; Niehof et al., 2021). The method combines QIAzol‐ and guanidinium‐based lysis with high‐salt and glycogen‐assisted RNA precipitation (Langwiński et al., 2024), together with acidic phenol‐chloroform‐isoamyl alcohol phase separation and subsequent magnetic bead–based purification using the MagMAX™ mirVana™ Total RNA Isolation Kit (Niehof et al., 2021).

**Figure 5:**
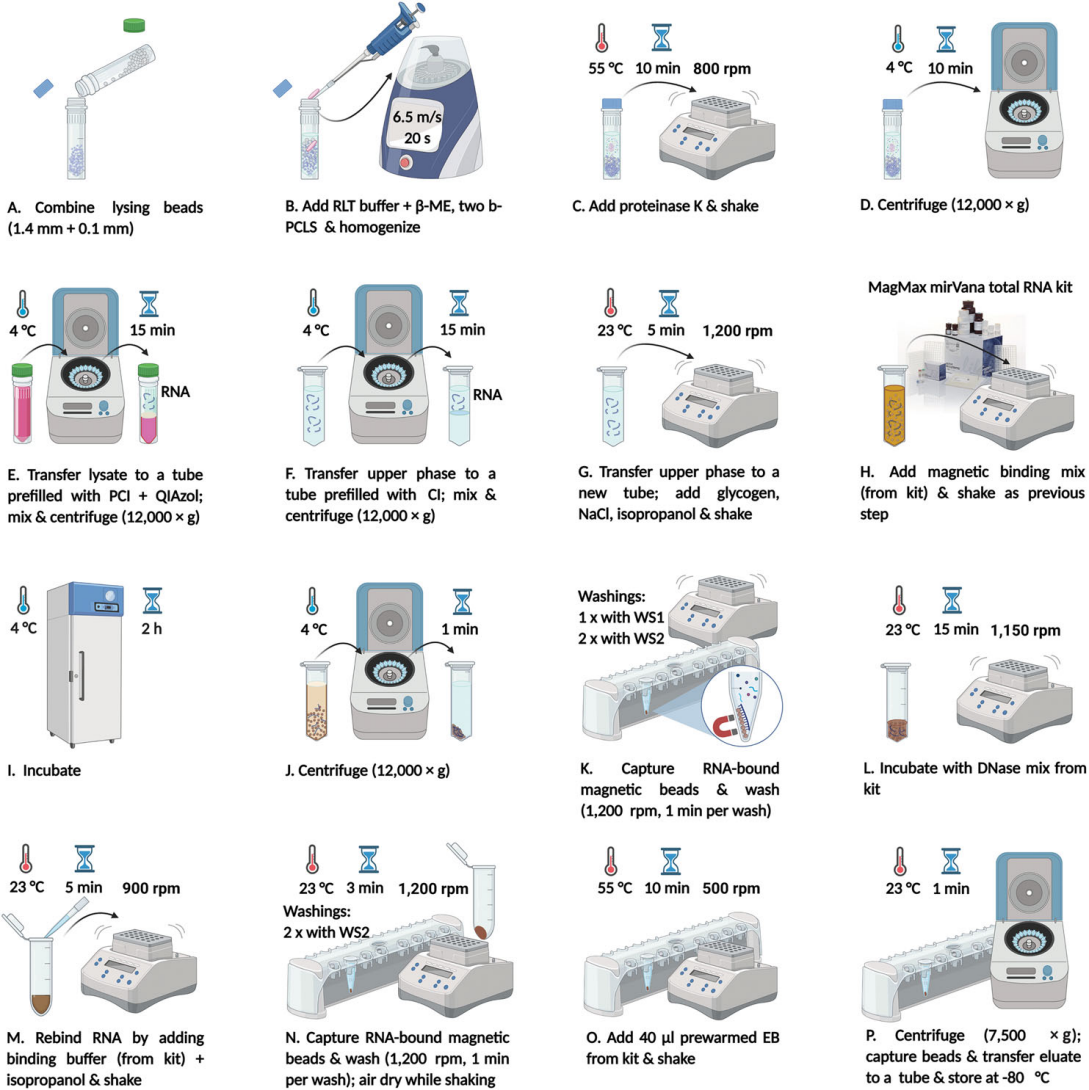
Schematic representation of the integrated RNA extraction workflow optimized for agarose‐inflated bovine precision‐cut lung slices (b‐PCLS) described in Basic Protocol 4. The procedure combines mechanical homogenization and chemical lysis using RLT lysis buffer supplemented with β‐mercaptoethanol (β‐ME) (A‐B), followed by proteinase K digestion (C‐D). Sequential organic phase separation is performed using phenol‐chloroform‐isoamyl alcohol (PCI) in combination with QIAzol (E), followed by secondary chloroform‐isoamyl alcohol (CI) extraction (F). RNA is subsequently recovered using high‐salt [NaCl (1.2 M)], isopropanol, and glycogen‐assisted precipitation, and purified with magnetic beads from the MagMAX™ mirVana™ Total RNA Isolation Kit (G‐J). RNA‐bound beads are washed with washing solutions 1 and 2 (WS1 and WS2) (K), treated with DNase (L), re‐bound (M), washed again (N), and finally eluted using elution buffer (EB) (O‐P).

Modifications introduced during integration include the combined use of different bead sizes and optimized homogenization parameters to improve mechanical disruption of agarose‐containing tissue; incorporation of proteinase K digestion and additional clarification centrifugation steps to reduce agarose and protein carryover; and increased magnetic bead input in the presence of NaCl, glycogen, and isopropanol to enhance RNA‐binding efficiency.

Collectively, these adaptations enable efficient recovery of high‐quality, intact RNA from agarose‐inflated lung tissue suitable for downstream applications, including RT‐qPCR and host and bacterial gene expression analysis.

#### RNA extraction from b‐PCLS

##### Timing: ∼6 hr

All steps should be performed using RNase‐free consumables and reagents, and all work surfaces should be treated with RNase AWAY™ to prevent RNase contamination.

Steps involving phenol/chloroform, QIAzol, and isopropanol precipitation (steps 1–29) must be performed in a chemical fume hood. Subsequent washing and bead‐based purification steps may be carried out on the bench.

#### Tissue homogenization and lysis (Fig. 5A–D)

1 Thaw b‐PCLS samples on ice.
2 Transfer the contents of a 2‐mL tube containing 1.4‐mm Lysing Matrix D beads into a 2‐mL BeadBug tube containing 0.1‐mm zirconium beads to combine both bead sizes in a single tube (Fig. 5A).
  Using two bead sizes improves tissue disruption and facilitates the release of host‐ and pathogen‐associated RNA.
3 Prepare RLT lysis buffer supplemented with β‐mercaptoethanol (10:1, *v/v*). For six samples, mix 3 mL of RLT with 30 µL of β‐mercaptoethanol (β‐ME).
  RLT buffer contains guanidine isothiocyanate for cell lysis, while β‐ME inactivates RNases.
4 Add 500 µL of RLT‐β‐ME mixture to each mixed‐bead‐containing tube and keep on ice.
5 Transfer two b‐PCLS per tube; keep on ice until homogenization (Fig. 5B).
6 Homogenize samples using a FastPrep‐24™ 5G instrument (20 s at 6.5 m/s) or a FastPrep‐96 (15 s at 1,800 rpm).
7 Place tubes on ice for 2 min.
8 Incubate homogenates at RT for 5 min to promote dissociation of nucleoprotein complexes.
9 Add 480 µL RNase‐free water and 20 µL Proteinase K to each sample (Fig. 5C).
  Proteinase K digestion improves RNA recovery and purity by degrading residual proteins and nucleoprotein complexes in agarose‐inflated lung tissue. As a result, it enhances lysis, facilitates efficient phase separation, and improves accessibility for downstream DNase treatment.
10 Incubate at 55°C while shaking at 800 rpm for 10 min.
11 Centrifuge at 12,000 × g and 4°C for 10 min to pellet insoluble debris (Fig. 5D).
  This clarification step improves RNA purity in samples containing high levels of fat, protein, polysaccharides, or extracellular matrix components.

#### Phase separation (Fig. 5E–F)

12 Add 800 µL acidic phenol/chloroform/isoamyl alcohol (PCI, 25:24:1) and 100 µL QIAzol to the empty Lysing Matrix D tubes from step 2.
13 Transfer the cleared supernatant (∼900–1,000 µL) from step 11 into this tube.
14 Shake by hand for 30 s.
15 Centrifuge at 12,000 × g and 4°C for 15 min (Fig. 5E).
16 Carefully transfer the upper aqueous phase (∼800–900 µL) to a 2‐mL RNase‐free tube, avoiding the interface.
17 Add an equal volume of chloroform/isoamyl alcohol (CI) (49:1).
18 Shake by hand for 30 s and incubate at room temperature for 3 min.
19 Centrifuge at 12,000 × g and 4°C for 15 min (Fig. 5F).
20 Carefully transfer the upper aqueous phase (∼700 µL) to a 2‐mL RNase‐free tube, avoiding the interface.

#### RNA precipitation with glycogen, NaCl, isopropanol, and magnetic beads (Fig. 5G–I)

21 Add 1.5 µL glycogen (30 µg/sample) to the aqueous phase.
  Glycogen acts as a coprecipitant to improve RNA recovery.
22 Add 5 M NaCl to reach a final concentration of 1.2 M in the total precipitation volume (including isopropanol). Mix gently by hand for 60 s.
  Elevated salt concentration facilitates dissolution of LMP agarose present in b‐PCLS.
23 Add 400 µL of 100% isopropanol and mix gently for 60 s.
24 Prepare a premix of 20 µL magnetic beads and 10 µL of binding enhancer per sample (MagMAX™ mirVana Total RNA Kit).
25 Add 30 µL of the premix to each sample (Fig. 5H).
  The MagMAX™ mirVana Total RNA Kit recommends 10 µL magnetic beads per sample. Increasing the bead volume can improve RNA recovery and yield higher RNA concentrations in agarose‐containing PCLS.
26 Shake for 5 min at 1,200 rpm and 23°C (Fig. 5G).
27 Incubate at 4°C for 2 hr to allow RNA precipitation and binding to magnetic beads (Fig. 5I).

#### DNase treatment and bead‐based RNA purification (Fig. 5J–M)

28 Centrifuge samples at 12,000 × g and 4°C for 1 min to pellet beads (Fig. 5J).
29 Place microtubes on a magnetic stand to capture beads and carefully discard the supernatant (Fig. 5K).
30 Perform washing steps according to the MagMAX™ mirVana Total RNA Kit protocol (Fig. 5K):
  150 µL Wash Solution 1 (WS1) once
  400 µL Wash Solution 2 (WS2) twice
    The volume of Wash Solution 2 is increased compared to the kit protocol because the procedure is performed manually in microtubes rather than in 96‐well plates. This modification improves contaminant removal from agarose‐containing PCLS samples in microtubes.
31 After adding each wash solution, gently flick the tube with a finger to detach magnetic beads that may be stuck together.
  If beads remain clumped, gently tap the pellet with a pipette tip without pipetting to break up aggregates.
32 Mix the magnetic beads in each wash solution using a thermomixer at 1,200 rpm and 23°C for 1 min (Fig. 5K).
33 Centrifuge briefly at 7,500 × g for 15 s.
34 Place microtubes on a magnetic stand and carefully remove the supernatant.
35 After the final wash (WS2), add 50 µL of DNase mix (2 µL DNase + 48 µL DNase buffer) per sample (Fig. 5L).
  The DNase mix can be prepared in advance. Ensure that the RNA‐bound beads are fully resuspended in the DNase mix by gently pipetting up and down until completely dispersed. This maximizes DNA removal efficiency.
36 Incubate the microtubes for 15 min at 23°C while shaking at 1,150 rpm (Fig. 5L).
37 Rebind RNA by adding 50 µL of binding buffer, followed by 100 µL of isopropanol. Do not premix (Fig. 5M).
38 Mix for 5 min at 23°C while shaking at 900 rpm (Fig. 5M).
39 Perform two rounds of washing with 200 µL of WS2 (Fig. 5N).
40 Repeat steps 31–34 for each wash.
41 After removing the final supernatant, air‐dry the beads for 3 min while shaking at 1,200 rpm and 23°C (Fig. 5N).

#### RNA elution (Fig. 5O–P)

42 Add 40 µL of prewarmed Elution Buffer (EB) (40°C) to the beads. Pipette up and down.
43 Incubate for 10 min at 55°C, while shaking at 500 rpm (Fig. 5O).
44 Centrifuge at 7,500 × g for 1 min.
45 Capture the beads on a magnetic stand and transfer the eluate to a new RNase‐free tube (Fig. 5P).
46 Quantify RNA concentration using a Qubit fluorometer with the Broad‐Range RNA kit, assess purity using a NanoDrop spectrophotometer, and evaluate RNA integrity using a TapeStation system with the appropriate kit, following the manufacturers’ instructions (Tables 5 and 6).

**Table 5:** RNA quality metrics for total RNA extracted from bovine precision‐cut lung slices following *Pasteurella multocida* inoculation. Three biological replicates (animals) were included. Within each biological replicate, technical replicates consisted of two pooled slices per RNA extraction. RNA quantity was measured using a Qubit fluorometer. RNA purity was assessed by NanoDrop, with 260/280 ratios indicating protein contamination and 260/230 ratios indicating other contaminants, including salts and phenol. RNA integrity was determined using TapeStation to generate the RNA integrity number (RIN).

	Biological replicate (Animal)	Control negative replicate 1	Control negative replicate 2	Control negative replicate 3	Infected replicate 1	Infected replicate 2	Infected replicate 3
**Qubit concentration (ng/µL)**	1	11.8	24.1	10.4	23.4	45.1	15.1
	2	32.6	24.8	27.6	31.2	35.7	28.3
	3	19.6	16.2	12.4	23.6	24	17
**260/280**	1	1.5	1.7	1.5	1.7	1.8	1.7
	2	1.7	1.6	1.6	1.7	1.7	1.7
	3	1.6	1.6	1.6	1.6	1.7	1.6
**260/230**	1	0.8	0.7	0.2	1	1	0.6
	2	1	0.9	0.9	0.7	0.8	0.8
	3	0.8	1	0.8	0.7	1.2	1.1
**RIN value**	1	9.2	8.7	8.1	9.8	9	9.5
	2	9.9	9.7	9.8	8.5	8.8	9.2
	3	8.6	9.9	9.2	10	9.4	9.3

**Table 6:** RNA quality metrics for total RNA extracted from bovine precision‐cut lung slices (b‐PCLS) following bovine coronavirus (BCoV) inoculation and confirmation of viral replication. Three biological replicates (animals) were included. Within each biological replicate, technical replicates consisted of two pooled slices per RNA extraction. RNA quantity was measured using a Qubit fluorometer. RNA purity was assessed by NanoDrop, with 260/280 ratios indicating protein contamination and 260/230 ratios indicating other contaminants, including salts and phenol. RNA integrity was determined using TapeStation to generate the RNA integrity number (RIN).

	Biological replicate (Animal)	Control negative replicate 1	Control negative replicate 2	Control negative replicate 3	Infected replicate 1	Infected replicate 2	Infected replicate 3
**Qubit concentration (ng/µL)**	1	29.2	67.5	109	94.3	70.3	84.1
	2	49.2	24.1	35.6	44.2	72.9	49.2
	3	21.4	22.5	59.2	21.5	55.3	36.8
**260/280**	1	1.6	1.9	2	2	1.9	1.9
	2	1.7	1.6	1.6	1.6	1.7	1.6
	3	1.6	1.6	1.7	1.6	1.7	1.6
**260/230**	1	0.7	0.2	0.3	0.5	0.3	0.4
	2	1	0.7	0.8	0.8	1.1	1
	3	0.9	0.6	0.9	0.8	1	1.1
**RIN value**	1	9.3	8.3	9.8	9.9	9.8	9.7
	2	9.5	9.1	9.3	9.2	8.9	9
	3	9	9.3	8.8	9.4	10	9.7

47 Store RNA at −80°C until downstream applications.

## Commentary

### Critical Parameters

The preparation and experimental use of bovine precision‐cut lung slices (b‐PCLS) require prior training and rigorous standardization, which may be challenging for inexperienced users. Several methodological factors are critical for ensuring tissue viability, experimental reproducibility, and high‐quality downstream readouts:

• **Vibratome and slicing workflow**: Performance varies between vibratome systems. When using a Compresstome, several steps—such as punching tissue cores, mounting the cores with superglue, slicing, and slice collection—can be performed in parallel. Assistance during these steps is strongly recommended to maintain both speed and consistency.

• **Time from slaughter to processing**: Minimizing the interval between animal slaughter and lung inflation with the RPMI–agarose mixture is crucial. Lungs should be processed within 1 hr post‐slaughter and kept on ice throughout handling to preserve tissue integrity and cellular viability.

• **Bronchus cannulation**: Gentle insertion of the cannulation needle along the bronchial lumen is required to avoid parenchymal damage. Avoid excessive force during cannulation or inflation to prevent rupture of distal airways.

• **Slice thickness**: Uniform slice thickness is critical for reproducibility. Slices should generally be ≤500 µm to ensure homogeneous nutrient diffusion, consistent exposure to treatments or infectious agents, and comparable responses across replicates and experiments.

• **RNA extraction**: Agarose inflation of lung tissue presents a significant challenge for RNA recovery. Pooling slices (two slices per sample in this protocol) is recommended to obtain sufficient RNA for downstream applications, such as transcriptomic analyses. Efficient homogenization, rigorous phase separation, and optimized precipitation steps are essential to maximize RNA yield and integrity.

• **Interpretation of experimental findings in the context of BRD pathogenesis**: While b‐PCLS preserves native lung architecture and cellular heterogeneity, including resident alveolar macrophages, dendritic cells, epithelial cells, and the extracellular matrix, it does not support active recruitment of circulating immune populations, such as neutrophils and monocytes, nor the systemic inflammatory mediators that contribute to BRD immunopathology *in vivo* (Eymieux et al., 2024; Fletcher Wheeler et al., 2026; Koziol‐White et al., 2024; Liu et al., 2019; Viana et al., 2022). This limits the model's capacity to recapitulate recruited innate immune responses and systemic adaptive immunity but conversely allows the study of resident cell responses. In addition, the bronchoalveolar lining fluid, including soluble pattern recognition molecules and chemokine gradients, is partially disrupted during tissue preparation, and cytokine concentrations measured in culture supernatants may not directly reflect *in vivo* levels.

• **Viability**: The viable culture period of PCLS can extend to 7–15 days, depending on species and culture conditions (Preuß et al., 2022). Nevertheless, functional changes occur over time, including decreased cytokine responsiveness and a gradual decline in ciliary beat (Neuhaus et al., 2017; Preuß et al., 2022). Experimental endpoints should therefore be selected with reference to the viability window of the protocol used, and prolonged culture should be accompanied by systematic viability monitoring.

• **Donor variability**: Experimental outcomes may be influenced by donor characteristics, including age, health status, and breed, which can affect baseline tissue quality, immune cell composition, and responsiveness to stimulation (Koziol‐White et al., 2024; Liu et al., 2019; Neuhaus et al., 2017). In bovine tissue specifically, subclinical respiratory conditions or breed‐associated differences in immune responses may introduce variability between donors that is not always apparent at the time of tissue collection. To account for this, biological replicates collected from separate donor animals are required, and results should be interpreted in the context of inter‐donor variability.

**Sequential infection model**: The individual inoculation protocols described here do not recapitulate the sequential polymicrobial pathogenesis of BRD, in which virus‐induced disruption of local defenses precedes bacterial invasion of the lower airways (Storoni et al., 2026). However, sequential co‐infection protocols are readily feasible in PCLS and have been described in the literature (Gaudino et al., 2023; Krimmling & Schwegmann‐Weßels, 2017; Vötsch et al., 2021), representing a logical extension of the workflows presented here. Overall, PCLS infection data should be interpreted as mechanistic evidence of discrete tissue‐level host–pathogen interactions rather than as a direct model of disease under field conditions.

### Troubleshooting

Careful attention to the parameters outlined above is essential for successful b‐PCLS generation and RNA extraction (see also Table 7).

**Table 7:** Troubleshooting guide for bovine precision‐cut lung slices (b‐PCLS) generation and downstream processing

Step	Problem	Possible reason	Solution
Lung inflation	Uneven or fragile slices	Incomplete agarose inflation; air bubbles	Ensure uniform lung inflation prior to agarose solidification; avoid introducing air bubbles during cannulation
Mounting tissue cores onto the specimen tube	Tissue cores slip or deform	Wet tissue cores in RPMI; overcrowding; excessive pressure with tweezers	Blot tissue cores on absorbent paper before mounting; use multiple containers to avoid overcrowding the tissue cores; handle with blunt‐tip tweezers, applying minimal pressure.
Vibratome slicing	Variable slice thickness	Vibratome miscalibration; dull blade; incorrect cutting speed, amplitude, or thickness	Optimize cutting parameters; replace the blade regularly; collect only slices with uniform thickness
PCLS washing after generation	Residual agarose in bronchioles, reducing ciliary activity or pathogen adherence	Insufficient washing; use of cold DPBS	Wash slices multiple times with prewarmed DPBS; refresh culture medium every other day
Adherence assay	Variable CFU per slice	Heterogeneous bronchiole size or multiple bronchioles per slice	Standardize slices based on bronchiole number and size across replicates
Homogenization for RNA extraction	Incomplete tissue disruption; low RNA yield	Insufficient bead mixing or low speed	Slightly increase the homogenization speed or duration; verify bead size and composition
Homogenization for RNA extraction	RNA degradation	Over‐homogenization; sample warming	Limit homogenization cycles or durations; keep samples on ice between runs
Phase separation during RNA extraction	Aqueous phase contamination	Disturbance of interphase	Carefully aspirate the upper aqueous phase, leaving a small buffer volume above the interphase
RNA precipitation after NaCl, isopropanol, and magnetic bead incubation	Rigid pellet; magnetic bead aggregation; ineffective washing; poor DNase digestion	Prolonged centrifugation leading to bead compaction	Centrifuge briefly (≤1 min); gently tap bead pellet with a pipette tip to disperse beads without pipetting; avoid aspirating beads
DNase treatment	Residual genomic DNA	Incomplete DNase digestion	Ensure RNA‐bound beads are fully resuspended in DNase mix; use correct DNase concentration and incubation time
Final RNA quality	Low A260/230 ratio	Residual salts or phenol contamination	Increase wash stringency; ensure complete removal of ethanol and wash buffers before elution

### Understanding Results

The b‐PCLS generated in this study were obtained from the lungs of three freshly slaughtered cattle.

#### Expected tissue viability

**Figure 6:**
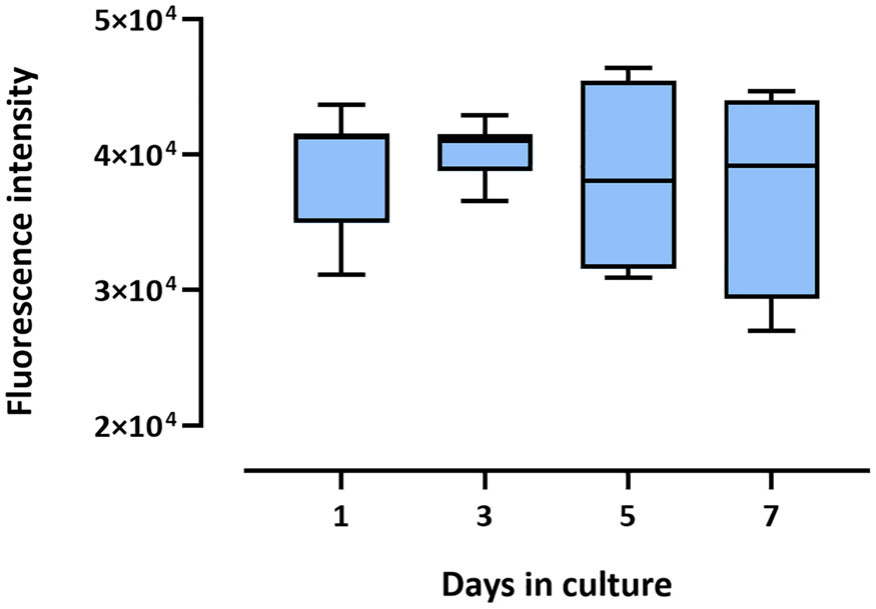
Metabolic activity indicating viability of bovine precision‐cut lung slices (b‐PCLS) over 7 days in culture post‐generation, assessed using the PrestoBlue high‐sensitivity cell viability reagent. Fluorescence intensity was quantified using a microplate reader. Data were obtained from three biological replicates (individual cattle), each analyzed in three technical replicates. The central line within each box represents the median, boxes represent the 25^th^–75^th^ percentiles, and whiskers indicate the minimum and maximum values.

Under the defined maintenance conditions, successful b‐PCLS preparation yields slices that remain viable and metabolically active for at least one week. During this period, ciliary activity is preserved without detectable decline, accompanied by a stable, high metabolic activity rate (Fig. 6).

Following preparation, each b‐PCLS is scored based on bronchiolar ciliary activity. On average, approximately 40% to 50% of slices exhibit ≥80% ciliary activity and correspondingly high metabolic activity, indicating robust viability. Approximately 10% to 15% of slices display no detectable ciliary activity (0%) and are considered non‐viable. The remaining slices demonstrate activity levels between 10% and 80%.

#### Anticipated infection dynamics

**Figure 7:**
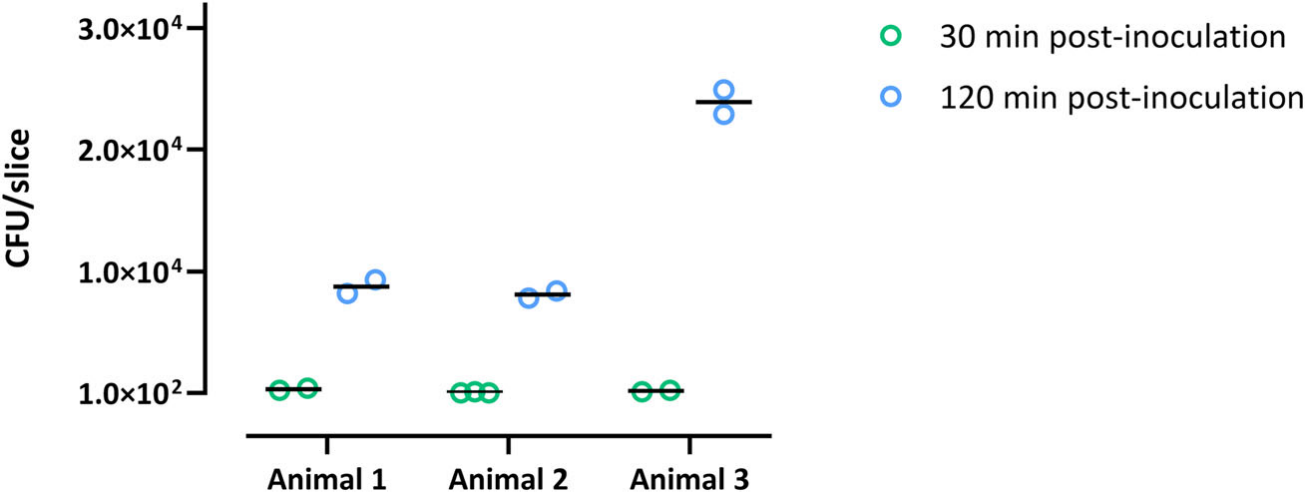
Adherence of *Pasteurella multocida* to the bronchial epithelial cell layer of bovine precision‐cut lung slices (b‐PCLS), expressed as colony‐forming units (CFU) per slice at 30 min and 120 min post‐inoculation. Data represent three biological replicates (individual cattle), each analyzed in two technical replicates with individual values shown as circles. Horizontal lines indicate the mean.

Following inoculation of highly active slices (≥80% ciliary activity) with *Pasteurella multocida*, slices can be monitored up to the desired experimental time point. In the present study, no reduction in ciliary activity was observed within 3 hr post‐inoculation (Video 3). However, homogenization and plating of the inoculated slices onto blood agar plates revealed viable, adherent, and/or invasive bacteria associated with the epithelial layer. Bacterial counts increased between 30 min and 2 hr post‐inoculation, indicating progressive bacterial–tissue interaction (Fig. 7).

**Figure 8:**
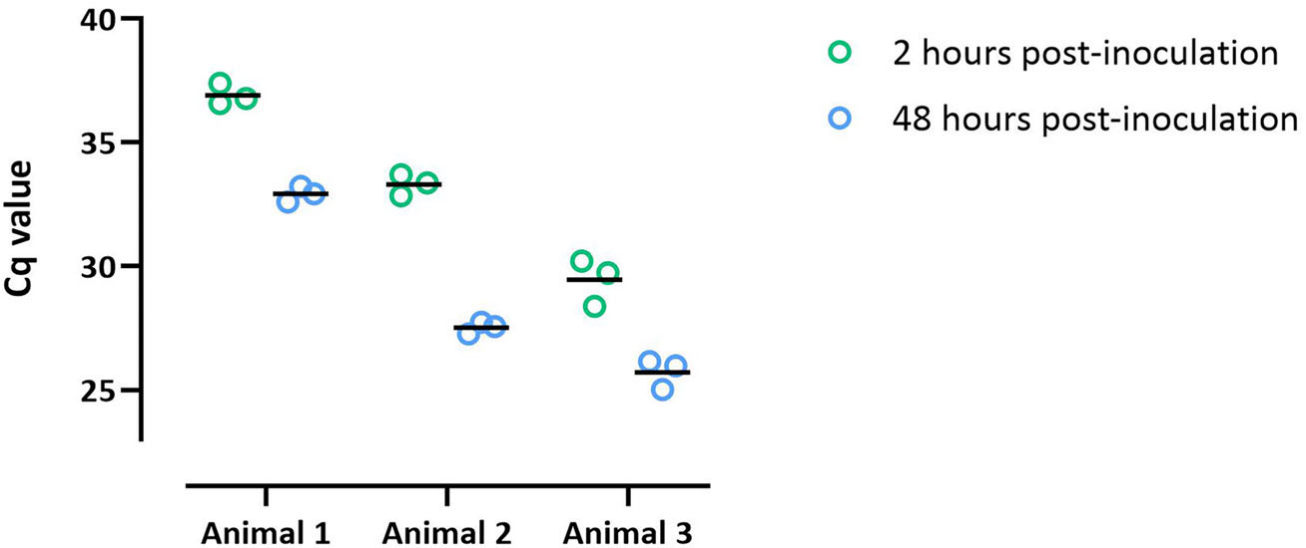
RT‐qPCR Cq values for bovine coronavirus (BCoV) detected in supernatants from bovine precision‐cut lung slices (b‐PCLS) at two time points post‐inoculation. The 2 hr time point corresponds to the time immediately after the 2 hr viral adsorption period, following removal of the viral inoculum, washing of the slices, addition of fresh maintenance medium, and subsequent collection of supernatants for RNA extraction. The second time point represents 48 hr post‐inoculation. Data represent three biological replicates (individual cattle), each with three technical replicates (circles). Lines indicate the mean.

B‐PCLS inoculated with BCoV can be maintained under the defined culture conditions and monitored for the desired experimental duration. In the present study, slices were followed up to 48 hr post‐inoculation. Viral RNA extracted from culture supernatants at 48 hr post‐inoculation, compared to the 2 hr time point, showed a decrease in Cq values, consistent with active viral replication over time (Fig. 8). Despite this increase in viral replication, no decline in ciliary activity was observed over the same period, indicating preserved epithelial function.

#### RNA quality and downstream applications

RNA extraction from non‐inoculated and *P. multocida*‐inoculated b‐PCLS confirmed the effectiveness of the modified isolation method. The integrated protocol consistently yielded RNA of sufficient quality and quantity for downstream applications, including host and Gram‐negative bacterial transcriptomic analyses (Table 5). In addition, total RNA extracted from BCoV‐inoculated and non‐inoculated slices met the quality criteria required for downstream host gene expression analyses (Table 6). Although the 260/230 ratios were below optimal, the extracted RNA supported further applications, as demonstrated by its successful use in RNA sequencing‐based transcriptomic analyses in parallel studies.

### Time Considerations

All timing estimates in this article are based on generating up to 72 bovine precision‐cut lung slices (b‐PCLS) in a single preparation. Bacterial infection experiments include follow‐up periods of up to 3 hr post‐inoculation, while viral infection experiments extend up to 48 hr post‐inoculation. Timing for total RNA extraction from b‐PCLS is based on processing six replicates, with two slices pooled per replicate. Actual time requirements may vary depending on the operator's experience, the availability of assistance during slicing, and the complexity of the experimental design.

Estimated durations for each Basic Protocol are as follows:

• **Basic Protocol** **1**: 2 days (Day 1: b‐PCLS generation; Day 2: culture medium change, followed by ciliary activity and metabolic activity assessment)

• **Basic Protocol** **2**: 4 days (Days 1–3: bacterial culture preparation; Day 4: inoculation, incubation, and sampling)

• **Basic Protocol** **3**: 2 days (viral inoculation, incubation, and sampling)

• **Basic Protocol** **4**: 2 days (Day 1: viral RNA extraction from supernatants and RT‐qPCR; Day 2: RNA extraction from b‐PCLS)

### Author Contributions

A.A. learned the PCLS technique at the University of Veterinary Medicine Hannover and adapted/established it locally with modifications. A.A. also performed infection experiments and RNA extractions, conducted formal analyses, and drafted the manuscript. J.M. provided training and guidance on the PCLS technique, bacterial infection experiments, and RNA extraction. G.A., V.S.O., and A.K.L. contributed to the bacterial infection experiments. A.K. and V.G.W. contributed to the viral infection experiments. V.G.W. contributed to viral RNA extractions. A.K.L., M.M., and V.S.O. secured funding. A.K.L and M.M. designed and supervised the experiments. All authors contributed to the manuscript revision and approved the final version.

### Conflict of Interest

The authors declare no conflict of interest.

## Data Availability

The authors can provide original data upon request.
